# Connexin43 Hemichannel Targeting With TAT-Gap19 Alleviates Radiation-Induced Endothelial Cell Damage

**DOI:** 10.3389/fphar.2020.00212

**Published:** 2020-03-05

**Authors:** Raghda Ramadan, Els Vromans, Dornatien Chuo Anang, Ines Goetschalckx, Delphine Hoorelbeke, Elke Decrock, Sarah Baatout, Luc Leybaert, An Aerts

**Affiliations:** ^1^Radiobiology Unit, Belgian Nuclear Research Centre (SCK•CEN), Mol, Belgium; ^2^Department of Fundamental and Basic Medical Sciences, Physiology Group, Ghent University, Ghent, Belgium; ^3^Centre for Environmental Health Sciences, Hasselt University, Hasselt, Belgium; ^4^Biomedical Research Institute and Transnational University of Limburg, Hasselt University, Hasselt, Belgium; ^5^Protein Chemistry, Proteomics and Epigenetic Signaling Group, Faculty of Pharmaceutical, Biomedical and Veterinary Sciences, University of Antwerp, Antwerp, Belgium; ^6^Department of Molecular Biotechnology, Ghent University, Ghent, Belgium

**Keywords:** atherosclerosis, endothelial damage, ionizing radiation, connexin43 hemichannels, TAT-Gap19

## Abstract

**Background:**

Emerging evidence indicates an excess risk of late occurring cardiovascular diseases, especially atherosclerosis, after thoracic cancer radiotherapy. Ionizing radiation (IR) induces cellular effects which may induce endothelial cell dysfunction, an early marker for atherosclerosis. In addition, intercellular communication through channels composed of transmembrane connexin proteins (Cxs), i.e. Gap junctions (direct cell-cell coupling) and hemichannels (paracrine release/uptake pathway) can modulate radiation-induced responses and therefore the atherosclerotic process. However, the role of endothelial hemichannel in IR-induced atherosclerosis has never been described before.

**Materials and Methods:**

Telomerase-immortalized human Coronary Artery/Microvascular Endothelial cells (TICAE/TIME) were exposed to X-rays (0.1 and 5 Gy). Production of reactive oxygen species (ROS), DNA damage, cell death, inflammatory responses, and senescence were assessed with or without applying a Cx43 hemichannel blocker (TAT-Gap19).

**Results:**

We report here that IR induces an increase in oxidative stress, cell death, inflammatory responses (IL-8, IL-1β, VCAM-1, MCP-1, and Endothelin-1) and premature cellular senescence in TICAE and TIME cells. These effects are significantly reduced in the presence of the Cx43 hemichannel-targeting peptide TAT-Gap19.

**Conclusion:**

Our findings suggest that endothelial Cx43 hemichannels contribute to various IR-induced processes, such as ROS, cell death, inflammation, and senescence, resulting in an increase in endothelial cell damage, which could be protected by blocking these hemichannels. Thus, targeting Cx43 hemichannels may potentially exert radioprotective effects.

## Introduction

Adjuvant radiotherapy is a standard therapy for breast cancer treatment after conservative surgery and mastectomy (Yang and Ho, 2013). However, exposure of healthy tissue, in particular, the heart, to ionizing radiation (IR) often increases the risk for the development of cardiovascular diseases (CVD), especially atherosclerosis (Darby et al., 2003; Marks et al., 2005; Baker et al., 2011; Cutter et al., 2011; Yusuf et al., 2011; Darby et al., 2013; Aleman et al., 2014; Van Nimwegen et al., 2015; Baselet et al., 2016). Although modern radiotherapy techniques reduce the volume of the heart and major coronary vessels exposed to high doses of IR, some exposure is often unavoidable, especially in the case of left-sided breast cancer, in which case the accumulative dose received by the heart area is in the order of ∼6.6 Gy (compared to 2.9 Gy for right-sided breast cancer) (Darby et al., 2013; Eftekhari et al., 2015; Al-Kindi and Oliveira, 2016; Cho et al., 2018). A population-based case-control study in women who underwent radiotherapy for breast cancer indicated a significant increase of 7.4% in the rates of major coronary events (i.e. myocardial infarction, coronary revascularization, or death from ischemic heart disease) per increase of 1 Gy in the cardiac exposure dose, without apparent threshold (Darby et al., 2013). In addition to breast cancer, exposure of the cardiovascular system to IR may occur during thoracic cancer radiotherapy such as head-and-neck cancer, Hodgkin’s lymphoma, and esophageal cancer (Borghini et al., 2013). Indeed, growing evidence indicates a link between IR exposure at high and medium doses (>0.5 Gy) and atherosclerosis development (Stewart et al., 2006; Hoving et al., 2008; Shimizu et al., 2010; Icrp et al., 2012; Darby et al., 2013; Kreuzer et al., 2015; Little, 2016; Cheng et al., 2017). However, the underlying molecular mechanisms remain not entirely understood. In addition, there is growing epidemiological evidence nowadays that indicates an excess risk for CVD at much lower IR doses than previously thought (<0.5 Gy) (Yamada et al., 2005; Kreuzer et al., 2015; Baselet et al., 2016; Little, 2016). However, these data are suggestive rather than persuasive due to a lack of statistical power and the fairly limited knowledge on the underlying molecular mechanisms. Hence, effects of radiation exposure on the cardiovascular system, especially at low dose exposure, are still not fully characterized, possibly resulting in improper radiation protection.

Exposure of endothelial cells to IR induces DNA damage and ROS which in turn induces oxidative stress, cellular Ca^2+^ overload, inflammation, senescence and apoptosis which may cause endothelial cell dysfunction, an early marker for atherosclerosis (Baker et al., 2011; Yusuf et al., 2011; Borghini et al., 2013; Madan et al., 2015; Baselet et al., 2016; Tapio, 2016). Intercellular communication mediated by gap junctions and hemichannels, may modulate endothelial response to IR, and therefore, the atherosclerotic process (Azzam et al., 2001; Little, 2006; Ohshima et al., 2012). Gap junctions permit passive diffusion of small hydrophilic molecules and ions of below ∼1.5 kDa molecular weight between adjacent cells. Gap junctions are usually open under physiological conditions, and they are important both for slow physiological processes (e.g. cell growth and proliferation) and fast activities such as electrical impulse conduction in the heart or communication between vascular endothelial cells to transmit upstream vasodilatory messages (De Wit and Griffith, 2010). In contrast to gap junctions, plasma membrane hemichannels are typically closed under physiological conditions (Orellana et al., 2011), and can open in response to several stress signals (e.g. mechanical stimulation, stress-associated stimuli such as ischemia, oxidative stress, a modest increase in intracellular Ca^2+^ or pro-inflammatory conditions) (Ramachandran et al., 2007; De Vuyst et al., 2009; Orellana et al., 2009; Johansen et al., 2011; Meunier et al., 2017). When open, hemichannels facilitate bidirectional passage over the plasma membrane of ions and signaling or metabolic molecules of below ∼1.5 kDa molecular weight (e.g. Ca^2^**^+^**, prostaglandin E2, glutathione, glutamate, ATP, NAD^+^, and others) (Wang et al., 2013a). Uncontrolled opening of hemichannels can result in loss of cell-essential metabolites, excessive entry of Na^+^ and Ca^2+^ and ATP leakage which in turn can lead to activation of apoptotic caspases, nitric oxide production, and inflammation (Decrock et al., 2009a, b; Saez et al., 2010; Ohshima et al., 2012; Decrock et al., 2017; Leybaert et al., 2017). These events may potentially trigger significant changes in cell homeostasis and cause cell dysfunction.

Gap junctions and hemichannels are composed of transmembrane connexin (Cx) proteins. Endothelial cells, the interior lining of blood vessels and cardiac valves, express three main Cx isotypes, namely Cx37, Cx40, and Cx43. Increasing evidence indicates that Cxs play a role in atherosclerosis progression (27, 29–31). Cx37 and Cx40 are suggested to have atheroprotective effects as it was reported that healthy endothelial cells have a widely distributed Cx37 and Cx40 expression pattern, while both Cxs are lost in the endothelium covering the advanced atherosclerotic plaques (Kwak et al., 2002; Morel, 2014). In contrast, Cx43 has been associated with atherogenesis as it is highly expressed in atherosclerotic plaques compared to control (Kwak et al., 2002; Morel, 2014). Cx43 also has atherogenic properties as decreasing Cx43 expression reduces the formation of atherosclerotic lesions *in vivo* (Kwak et al., 2003; Wong et al., 2003; Wang et al., 2013b) while *in vivo* Cx43 upregulation increased the expression of cell adhesion proteins such as VCAM-1, thereby enhancing monocyte-endothelial adhesion, a key event in initiating atherosclerosis (Yuan et al., 2015). Furthermore, Cx43 has been implicated in endothelial cellular stiffness that is associated with cardiovascular disease and atherosclerosis (Okamoto et al., 2017).

Besides the role of Cx in the development of atherosclerosis, it was previously reported that Cx expression is sensitive to ionizing radiation. Cx43 expression was increased in human skin fibroblast after exposure to 10 mGy of α-particles (Azzam et al., 2003). In addition, Cx43 expression is elevated in response to low dose gamma-ray exposure (^137^Cs source) in human neonatal foreskin fibroblast (Glover et al., 2003), in response to 5 Gy X-rays in mouse brain endothelial cell line bend3 (Banaz-Yasar et al., 2005) and in cardiac myocytes in an *in vivo* animal model upon exposure to high-LET radiation (Amino et al., 2010). Finally, it was reported that 0.5 Gy of gamma-rays exposure induced Cx43 hemichannels opening in B16 melanoma cells (Ohshima et al., 2012).

Although Cxs and their channels have been reported to be involved in the pathogenesis of atherosclerosis and to be sensitive to IR exposure, their role in radiation-induced endothelial cell response were never investigated (Azzam et al., 2003; Pfenniger et al., 2013). We previously demonstrated that single and fractionated IR induces acute and persistent upregulation of Cx43 gene and protein expression in the coronary artery and microvascular endothelial cells (Ramadan et al., 2019). In addition, we demonstrated that IR induces acute and long-lived Cx43 hemichannel opening in a dose-dependent manner (Ramadan et al., 2019). Here, we aimed at investigating the involvement of Cx43 hemichannels in endothelial cell responses induced by IR exposure, by making use of the Cx43-targeting peptide TAT-Gap19 (Abudara et al., 2014). TAT-Gap19 was previously reported to specifically block Cx43 hemichannels in different *in vitro* experimental models, as observed by ATP release and dye uptake assays (Abudara et al., 2014; Willebrords et al., 2017; Maatouk et al., 2018; Saez et al., 2018; Walrave et al., 2018). These findings were supported by electrophysiological measurements of Cx43 hemichannel unitary currents demonstrating that Gap19 inhibits Cx43 hemichannels in HeLa cells overexpressing Cx43 (Wang et al., 2013b; Gadicherla et al., 2017), in acutely isolated pig ventricular cardiomyocytes (Wang et al., 2013b) and in primary astrocytes (Freitas-Andrade et al., 2019). In depth mechanistic investigations based on surface plasmon resonance studies revealed a direct binding of Gap19 to the C-terminal tail of Cx43, thereby preventing the CL loop/CT tail interaction which is essential for Cx43 hemichannel activity, whereas closure of gap junctions is prevented (Ponsaerts et al., 2010; Iyyathurai et al., 2018). Linking Gap19 to the HIV-derived TAT internalization sequence promotes its membrane permeability and reduces the concentration needed for half-maximal Cx43 hemichannel inhibition by 5-folds. We found that TAT-Gap19 reduced intracellular ROS generation, cell death, inflammation, and premature cell senescence induced by IR in the immortalized human coronary artery and microvascular endothelial cells. Collectively, these results indicate a functional linkage between hemichannel opening and post-irradiation ROS, inflammation, and cell death/senescence responses.

## Materials and Methods

### Cell Culture

Two human endothelial cell lines: Telomerase Immortalized human Coronary Artery Endothelial cells (TICAE) from the European Collection of Authenticated Cell Cultures (ECACC), and Telomerase Immortalized human Microvascular Endothelial cells (TIME) from the American Type Cell Culture (ATTC), were grown in MesoEndo Cell Growth Medium (Sigma-Aldrich Co., LCC, Diegem, Belgium) at 37°C in a humidified incubator supplemented with 5% CO2. Cells were split every 3/4 days, with 0.05% trypsin supplemented with 0.02% ethylenediaminetetraacetic acid (EDTA). The passage number 28 until passage 36 was used in all the experiments. Moxi Z Mini Automated Cell Counter (ORFLO Technologies, Ketchum, ID, United States) was used to count the cells. Cells were not passaged after irradiation for all experiments.

### Irradiation

X-irradiation was performed at the Laboratory for Nuclear Calibrations (LNK) of the Belgian Nuclear Research Centre (SCK•CEN), in accordance to ISO 4037 and under ISO 17025 accreditation of LNK. TICAE and TIME cells were irradiated when reached 100% confluence with 0.1 and 5 Gy of single X-rays doses, at a dose rate of 0.5 Gy/minute with a vertical, point source X-ray beam using a Xstrahl 320 kV tube (tube voltage: 250 kV; filtration: 3.8 mmAl+ 1.4 mm Cu+ Dose Area Product (DAP) monitor ionizing chamber; tube current 12 ma) (Camberley, United Kingdom).

### Intracellular ROS Detection

In order to assess intracellular ROS production, IncuCyte live cell imaging was used to have fast imaging in a large number of replicates over multiple time points (Jeong et al., 2014). TICAE and TIME cells were seeded in 96-well plate in 16 replicates at a density of 10 000 cells/well. Three days later, cells reached 100% confluence. At 30 min before irradiation, a fresh MesoEndo Cell Growth Medium without phenol red (Sigma-Aldrich Co., LCC, Diegem, Belgium) supplemented with 10 μmol/L of the CM-H2DCFDA dye (Sigma-Aldrich Co., LCC, Diegem, Belgium) was added to the cells, with or without 100 μM TAT-Gap19 (YGRKKRRQRRRKQIEIKKFK) (Genosphere Biotechnologies, Paris, France). Fluorescence signals were measured at 45 min, 2 h and 3 h after irradiation [CM-H2DCFDA dye became saturated that might be due to prolonged laser illumination-induced cellular ROS production, or the dye leaked outside the cells after this time (Koopman et al., 2006)] using the Incucyte ZOOM^®^ system (Essen Bioscience, Ann Arbor, MI, United States), 10x objective and FITC filter. Phase contrast imaging was performed to count the cells. Positive control of 10 μmol/L tert-butyl hydroxyperoxide (tBHP) was added to the cells directly before imaging. Images were analyzed, making use of the software package provided by the manufacturer; we used a training set of images from different doses to define the image processing procedure. The so-called Top-Hat background subtraction method was applied by measuring the radius of the fluorescence object (30 μm), and adjust a threshold of 1.5 (above the local background fluorescent intensity level) (Kapellos et al., 2016; Gupta et al., 2018), as described in Incucyte Background Fluorescence Technical Note. These process definitions were applied for all conditions in TICAE and TIME cells.

### Cell Death Assessment

#### Annexin V and Caspase 3/7

TICAE and TIME cells were seeded in 96-well plates at a density of 10 000 cells/well in eight biological replicates. After 3 days, cells were at 100% confluence. At 30 min before X-ray exposure, cells were refreshed with 150 μl medium supplemented with Caspase 3/7 reagent (1:1000 dilution) and Annexin V reagent (1:200 dilution) (Essen Bioscience, United Kingdom) with or without 100 μM TAT-Gap19 (Genosphere Biotechnologies, Paris, France). Fluorescence signals were measured from 4 h until 100 h after irradiation by imaging every 2 h using the Incucyte ZOOM^®^ system (Essen Bioscience, Ann Arbor, MI, United States). Different channels (TRITC, FITC and phase contrast) and a 10x objective were used. Spectral unmixing set as 8% of red removed from green was applied. The Incucyte software’s processing definition was set to recognize red (Annexin V) and green (Caspase 3/7) stained cells, making use of the Top-Hat background subtraction method. The fluorescence signal was normalized to cell count.

#### Dextran Fluorescein Dye Uptake Assay

Dextran fluorescein, staining late apoptotic and necrotic cells, was used to validate apoptosis in TIME cells after irradiation. Cells were seeded in a 24-well plate at a density of 1.5 × 10^5^ cells/well in 6 biological replicates. Three days later, cells reached 100% confluence. Before X-ray exposure with 30 min, cells were refreshed with medium with or without 100 μM TAT-Gap19 (Genosphere Biotechnologies, Paris, France). At 6 and 72 h after irradiation, cells were washed twice with HBSS complemented with 25 mm HEPES (Sigma-Aldrich Co., LCC, Diegem, Belgium) (HBSS-HEPES). Afterward, the cells were incubated with 200 μM dextran fluorescein (10 kDa) (Life Technologies, Merelbeke, Belgium), dissolved in HBSS-HEPES. After 6 min, the staining was removed by washing six times with HBSS-HEPES. Finally, the fluorescence was measured using the IncuCyte ZOOM^®^ system (Essen Bioscience, Ann Arbor, MI, United States) by using FITC channel and phase contrast to count the cells using a 10x objective. The fluorescence signal was normalized to cell count.

### Cytokine Detection

TICAE and TIME cells were seeded in a 6-well plate at a density of 2.5 × 10^5^ cells/well in 5 to 6 biological replicates. Two to 3 days later, cells reached 100% confluence. At 30 min before X-ray exposure, cells were refreshed with medium with or without 100 μM TAT-Gap19 (Genosphere Biotechnologies, Paris, France). At 24, 48, 72 h, and 7 days after irradiation, the supernatant was collected. The medium was changed only for the 7 days time point experiment on the fourth day with fresh medium alone or with medium supplemented with 100 μM TAT-Gap19. For the simultaneous detection of multiple cytokines in the supernatants, the Magnetic Luminex^®^ Assay (R&D Systems, Minneapolis, Canada) was used following the manufacturer’s instructions. Briefly, supernatants, standards, and microparticles were incubated into a 96-well plate which was pre-coated with cytokine-specific antibodies. The immobilized antibodies were able to bind the cytokines of interest after 2 h incubation, then the plate was washed, and incubation with a biotinylated antibody cocktail specific to the cytokines of interest was performed for 1 h. A second wash was performed in order to remove the unbound biotinylated antibodies. Further, a streptavidin-phycoerythrin conjugate was added to each well to bind to the biotinylated antibodies. After a final wash, the microparticles were resuspended in buffer and read using the Luminex^®^ MAGPIX Analyzer (R&D systems, Minneapolis, MN, Canada).

In order to normalize to cell number, DAPI staining was performed for the 48 h after irradiation time point. Briefly, cells were washed with phosphate buffered saline (PBS) and fixed with 4% paraformaldehyde (PFA) for 15 min at room temperature. Afterward, cells were washed with PBS and stained with 1 μg/ml 4′,6-Diamidine-2′-phenylindole (DAPI) dissolved in 1x TBST, 0.005 g/v% TSA blocking powder (PerkinElmer, FP1012) (TNB). After 1 h, cells were washed with PBS and DAPI signals were visualized using the Nikon Eclipse Ti inverted wide-field epifluorescence microscope equipped with a 5× magnification dry objective (Plan Fluor, numerical aperture 0.6) and TE2000-E Nikon camera, controlled by the NIS Elements software. The intensity of the DAPI signals was determined with the FIJI software. Cell count for 24 h after irradiation experiment was performed by Moxi Z Mini Automated Cell Counter (ORFLO Technologies, Ketchum, ID, United States) after trypsinization. For the 72 h, and 7 days post-irradiation cell counts, we used Incucyte ZOOM^TM^ phase-contrast imaging based on 49 images taken per well with the 10x objective.

### Senescence Detection by CPRG Assay

TICAE and TIME cells were seeded in 96 well plates at a density of 10 000 cells/well in 16 replicates. Three days after, cells were at 100% confluent. At 30 min before X-ray exposure, the cells were refreshed with 150 μl medium with or without with 100 μM TAT-Gap19 (Genosphere Biotechnologies, Paris, France). For 7 and 9 days post irradiation experiments, the medium was refreshed once, while for 14 days post irradiation experiment the medium was refreshed twice with 200 μl medium alone or with medium supplemented with 100 μM TAT-Gap19. At 7, 9, and 14 days post irradiation, cell number was determined using an Incucyte ZOOM^®^ live cell analysis system and related software (Essen Bioscience, Hertfordshire, United Kingdom). Afterward, the senescence-associated β-galactosidase activity in the cells was determined using the chlorophenol red β-D-galactopyranoside (CPRG) assay, as described previously (Baselet et al., 2017). The cells were washed with PBS and lysed using M-PERTM buffer (Thermo Fisher Scientific, Asse, Belgium). Subsequently, 1 × CPRG substrate (2 mM Chlorophenol Red β-D-galactopyranoside in CPRG assay buffer containing 50 mm KPO4, 1 mM MgCl2, pH 6) (Sigma-Aldrich, Overijse, Belgium) was added to each well and the plates incubated for 18 h at 37°C without CO2. A Clariostar^®^ microplate reader (BMG Labtech, Temse, Belgium) was used to measure the absorbance at 570 nm.

### DNA Damage Detection

To detect DNA double strand breaks after IR, TICAE and TIME cells were seeded in Nunc^TM^ Lab-Tek^TM^ Chamber Slide (Thermo Fisher Scientific, Asse, Belgium) in eight replicates, at a seeding density of 50 000 cells/well. Two days later, cells reached 100% confluence. At 30 min before IR, a fresh medium was added to the cells with or without 100 μm TAT-Gap19. At 1 h post irradiation, cells were fixed with 2% PFA (Sigma-Aldrich Co., LCC, Diegem, Belgium) and permeabilized with 0.25% Triton X-100 (Sigma-Aldrich Co., LCC, Diegem, Belgium) in PBS. Cells were blocked for 1 h in 1% normal goat serum (Thermo Fisher Scientific, Asse, Belgium) in Tris-NaCl (Perkin Elmer, Brussels, Belgium). Thereafter, staining with 1/300 primary anti-gamma H2AX antibody (Merck-Millipore #05-636) and 1/1000 anti-TP53BP1 antibody (Novus Biologicals #NB100-304) was performed for 1 h at 37°C. After washing three times with PBS, secondary Alexa Fluor 488 and 568 antibodies (Life Technologies, Merelbeke, Belgium) were applied for 1 h at 37°C. Finally, the slides were mounted using prolong Diamond Antifade Mountant with DAPI (Life Technologies, Merelbeke, Belgium), and cells were visualized with an Eclipse Ti inverted wide-field epifluorescence microscope (Nikon, Brussels, Belgium) with a 20 × Plan Fluor objective (NA 0.6) and an Andor Ixon EMCCD camera, controlled by the NIS Elements software. A z-stack of nine planes axially separated by 1 μm was applied, and 16 fields were captured for each replicate. The analysis was performed with FIJI software using the CellBlocks.ijm script (De Vos et al., 2010; Schindelin et al., 2012).

### Statistical Analysis

Non-parametric two-tailed Mann–Whitney *T*-test was performed for the analysis of all the experiments, except for cell death experiment using live cell imaging, where two-way ANOVA analysis followed by Tukey test was used. Data are presented as mean ± standard error of the mean. Statistical significance was considered when *P* < 0.05. GraphPad Prism 5.01 (GraphPad Software Inc., La Jolla, CA, United States) was used in all the analysis and occasional exclusion of outlier data points were performed using Grubbs’ test.

## Results

### Radiation-Induced Oxidative Stress Is Suppressed by TAT-Gap19

**FIGURE 1:**
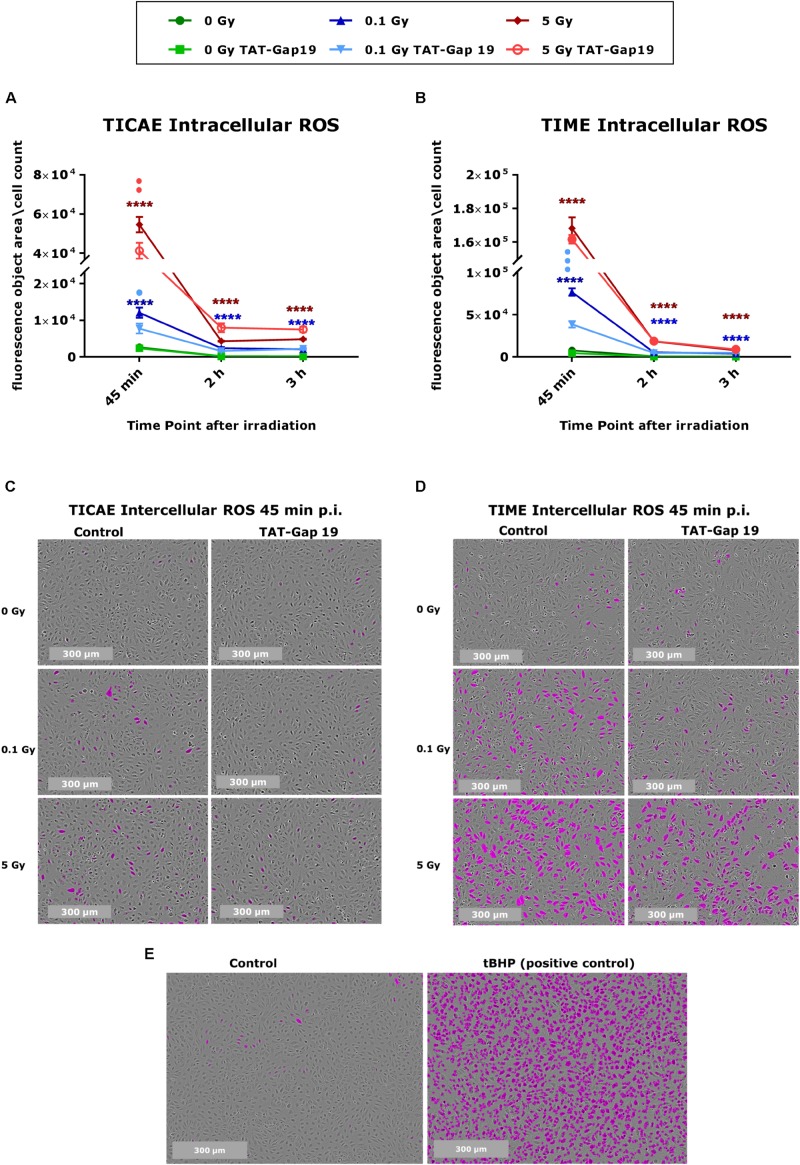
Radiation-induced ROS production and the effect of TAT-Gap19 in TICAE and TIME cells. Intracellular ROS was assessed using CM-H2DCFDA combined with Incucyte live cell imaging at three time points (45 min, 2 and 3 h) after irradiation **(A,B)**. **(C,D)** Representative images for ROS production at 45 min p.i. in TICAE and TIME cells, with purple colored cells indicating above threshold ROS signal. **(E)** Representative images for ROS production in TICAE cells in response to tert-Butyl hydroperoxide (tBHP) as a positive control condition; purple colored cells indicate above threshold ROS signal. The values represent the average ± SEM of 8–16 biological replicates for TICAE and TIME cells. Statistical analysis was done with a non-parametric Mann–Whitney *T*-test. *Indicates statistically significant differences compared to the respective 0 Gy controls. •Indicate statistically significant differences compared to the respective control condition (not treated with TAT-Gap19). */•*p* < 0.05; **/••*p* < 0.01; ***/•••*p* < 0.0001, ****/••••*p* < 0.00001.

Intracellular ROS production was measured in TICAE and TIME cells in response to exposure to 0.1 and 5 Gy X-rays, making use of Incucyte live cell imaging of CM-H2DCFDA fluorescence at 45 min, 2 h, and 3 h after IR exposure. In TICAE and TIME cells, a radiation-induced dose-dependent increase in intracellular ROS production was observed from 45 min until 3 h post irradiation (p.i.) (significant for 0.1 and 5 Gy), with the highest response at 45 min p.i. (Figures 1A–D). TIME cells showed to be more sensitive, as the extent of ROS production after 45 min of IR was 5-fold larger for the 0.1 Gy dose, and two times higher for the 5 Gy dose than in TICAE cells (Figures 1A–D). TAT-Gap19 significantly reduced ROS production at both doses (0.1 and 5 Gy) in TICAE cells at 45 min p.i., while in TIME cells, it significantly reduced ROS production only for the 0.1 Gy dose at 45 min p.i. (Figures 1A,B). Tert-Butyl hydroperoxide (tBHP) was used in the experiment as a positive control (Figure 1E).

### TAT-Gap19 Protects Against Radiation-Induced Cell Death

In order to investigate cell death after IR exposure, Annexin V (early and late apoptotic cells) and Caspase 3/7 activity (late apoptotic cells) were assessed in TICAE and TIME cells from 4 h until 100 h after IR using Incucyte live cell imaging. We furthermore tested the effect of hemichannel inhibition with TAT-Gap19.

**FIGURE 2:**
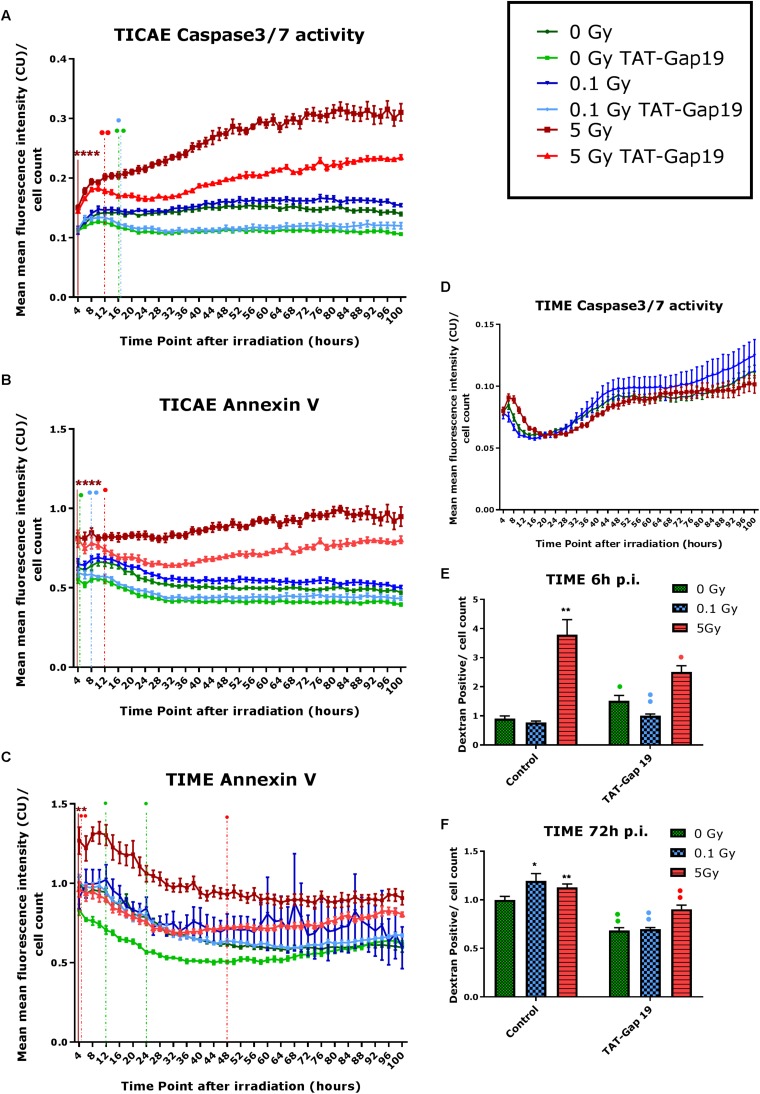
Radiation-induced cell death as assessed by Caspase 3/7 activity, Annexin V and 10 kDa dextran fluorescein staining, and effect of TAT-Gap19. **(A,D)** Caspase 3/7 activity in TICAE and TIME cells from 4 to 100 h p.i., **(B,C)** Annexin V level in TICAE and TIME cells from 4 to 100 h p.i. assessed by Incucyte live cell imaging of the mean fluorescence intensity measured in Calibrated Unit (CU) that takes into account acquisition time and fluorescent intensity. **(E,F)** Dextran fluorescein stained TIME cells at 6 and 72 h p.i. The values represent the average ± SEM of 6–8 biological replicates; statistical analysis was done with non-parametric two-way ANOVA followed by a Tukey test for Annexin V and Caspase 3/7 activity, and with a non-parametric Mann–Whitney *T*-test for the dextran fluorescein assay. *Indicates statistically significant differences compared to the respective 0 Gy controls. •Indicates statistically significant differences between the TAT-Gap19 group compared to the corresponding respective control conditions (not treated with TAT-Gap19). */•*p* < 0.05; **/••*p* < 0.01; ***/•••*p* < 0.0001, ****/••••*p* < 0.00001.

In TICAE cells, a significant increase in Caspase 3/7 activity and Annexin V was observed for the 5 Gy dose starting from 4 h and persistent until 100 h p.i., the last point of the recording (Figures 2A,B). TAT-Gap19 significantly reduced Caspase 3/7 and Annexin V for the 5 Gy dose starting from 12 h p.i. and persistent until the end of the recording (Figures 2A,B). In addition, TAT-Gap19 significantly reduced Caspase 3/7 activity for 0 and 0.1 Gy conditions starting from 16 h p.i, and persisting until the end (Figure 2A). TAT-Gap19 also significantly reduced Annexin V for the 0 Gy and 0.1 Gy conditions starting from 4 to 8 h on, respectively (Figure 2B).

In TIME cells, there was no significant change in Caspase 3/7 activity after 0.1 and 5 Gy of IR until 100 h p.i. (Figure 2D). However, a significant persistent increase in Annexin V level was observed at 5 Gy starting from 4 h p.i. until the end of the recording (Figure 2C). TAT-Gap19 significantly reduced Annexin V elevation for the 5 Gy dose in the 4–48 h p.i. time window. TAT-Gap19 also transiently reduced Annexin V for the 0 Gy condition (12–24 h p.i. time window; Figure 2C).

To further validate cell death in TIME cells, 10 kDa dextran fluorescein dye, which is known to enter and stain cells during necrosis or late apoptosis, was assessed at 6 and 72 h p.i. A radiation-induced increase in dextran fluorescein was observed for 5 Gy at 6 h p.i. and for both 0.1 and 5 Gy doses at 72 h p.i. (Figures 2E,F). These responses (including the control response) were inhibited by TAT-Gap19 (Figures 2E,F). An unexpected small increase in dextran fluorescein staining was observed with TAT-Gap19 for the control and 0.1 Gy conditions at 6 h p.i. (Figure 2E). We also evaluated the effect of TAT peptide alone (100 μm) on cell death in TICAE and TIME cells using Incucyte live cell imaging (Supplementary Figure 1). In TICAE cells, no significant changes in Caspase 3/7 activity and Annexin V were observed for TAT peptide from 4 h until 100 h after treatment (Supplementary Figures 1A,B). In TIME cells, it slightly increased Annexin V between 24 and 30 h after exposure (Supplementary Figure 1C).

### TAT-Gap19 Mitigates Radiation-Induced Inflammatory Responses

Different atherosclerosis inflammatory markers, selected based on a survey of available literature, were assessed in TICAE and TIME cells after 0.1 and 5 Gy of IR exposure at 24 h, 48 h, 72 h and 7 days p.i. We subsequently tested whether TAT-Gap19 affected these responses. The various inflammatory markers used are summarized in Supplementary Table 1. In total, we considered 12 different markers.

**FIGURE 3:**
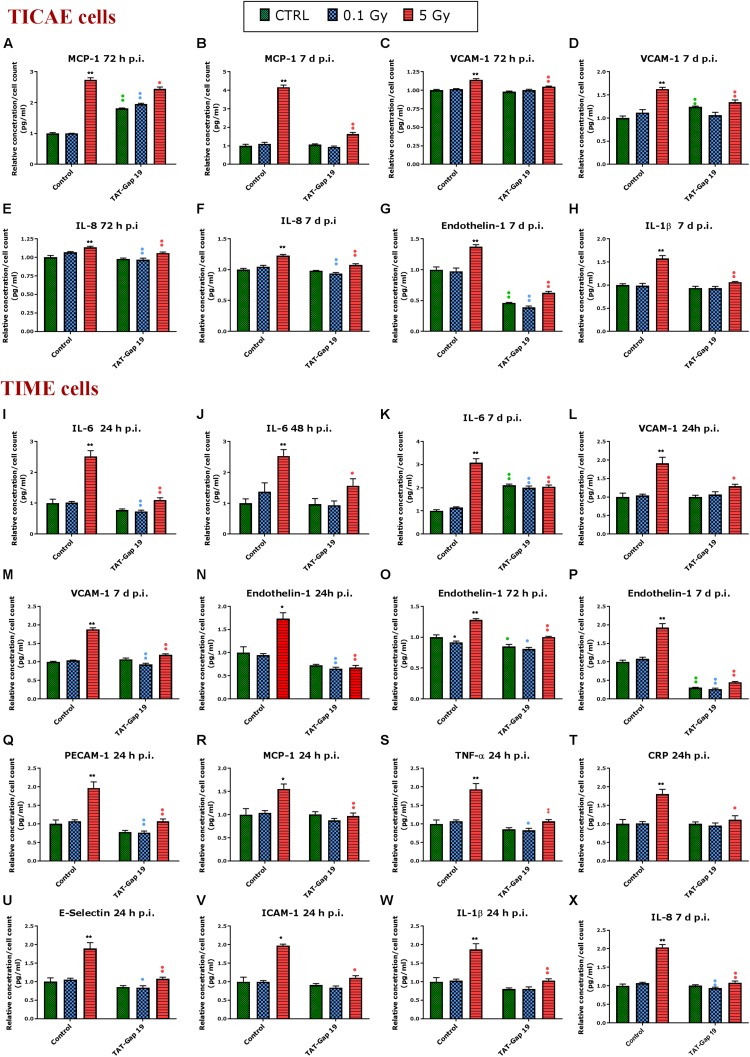
Radiation-induced inflammatory responses and the effect of TAT-Gap19. The response of various inflammatory markers in **(A–H)** TICAE cells and **(I–X)** TIME cells to 0.1 and 5 Gy irradiation conditions. The values represent the average ± SEM of 5–6 biological replicates; data were analyzed with a non-parametric Mann–Whitney *T*-test. *Indicates statistical differences compared to the respective 0 Gy controls. •Indicates statistically significant differences between the TAT-Gap19 group compared to the corresponding responses in the control group (not-treated with TAT-Gap19). */•*p* < 0.05; **/••*p* < 0.01. IL-1β, Interleukin 1 beta; IL-6, Interleukin 6; IL-8, Interleukin 8; ICAM-1, Intracellular adhesion molecule 1; VCAM-1, Vascular cell adhesion protein 1; PECAM-1, Platelet endothelial cell adhesion molecule-1; MCP-1, Monocyte chemotactic protein-1; TNF- α, Tumor necrosis factor alpha; CRP, C-reactive protein.

In general, IR exposure at a 5 Gy dose induced an increase in the majority of the markers (IL-6, MCP-1, PECAM-1, IL-1β, TNF-α, CRP, VCAM-1, E-Selectin, Endothelin-1, IL-8, and PAI-1) in both TICAE and TIME cells. TAT-Gap19 displayed variable effects, in some conditions decreasing while in others increasing the responses, especially in TICAE cells (Supplementary Table 1). In order to test the possibility that the viral TAT membrane translocation sequence could perhaps by itself elicit an effect, we selected several markers and evaluated their response to TAT peptide exposure at the 24 h time point, that corresponds to the time at which TAT-Gap 19 increased all tested markers in TICAE cells. We found that TAT (100 μm) significantly increased IL-6, MCP-1, IL-1β, and CRP in TICAE cells (Supplementary Figure 2). Notwithstanding these early (24 h) TAT side-effects on a subset of the markers in TICAE cells, TAT-Gap19 significantly reduced the responses of several markers in TICAE and TIME cells mostly at later time points. Figure 3 summarizes these responses for selected markers and time points, which are considered in the account that follows. In TICAE cells, MCP-1, VCAM-1, and IL-8 were significantly increased after 5 Gy IR exposure at 72 h and 7 days p.i., and TAT-Gap 19 significantly decreased these responses (Figures 3A–F). Radiation induced an increase in Endothelin-1 and IL-1β at 7 days p.i. in TICAE cells, and TAT-Gap 19 again significantly reduced these responses (Figures 3G,H).

In TIME cells, a radiation-induced significant increase in IL-6 at 5 Gy was observed at 24 h, 48 h, and 7 days p.i., and TAT-Gap19 significantly reduced these responses (Figures 3I–K). Next to that, radiation induced a significant increase in VCAM-1 for 5 Gy at 24 h and 7 days p.i. in TIME cells, and TAT-Gap19 again significantly reduced these responses (Figures 3L,M). Likewise, Endothelin-1 was significantly increased after 5 Gy irradiation in TIME cells at 24 h, 72 h and 7 days p.i., and TAT-Gap19 significantly decreased these responses (Figures 3N–P). Furthermore, TAT-Gap19 significantly reduced the increase of PECAM-1, MCP-1, TNF-α, CRP, E-Selectin, ICAM-1, and IL-1β at 24 h after 5 Gy irradiation in TIME cells (Figures 3Q–W). TAT-Gap19 also significantly decreased IL-8 levels after 5 Gy IR exposure at 7 days p.i. (Figure 3X) in TIME cells. In some of the experiments described above, TAT-Gap19 also had effects on the 0 Gy control and 0.1 Gy conditions in both cell types: it increased the MCP-1 control readout as well as the 0.1 Gy at 72 h p.i. measurement in TICAE cells (Figure 3A), and also the IL-6 control and 0.1 Gy at 7 days p.i. readouts in TIME cells (Figure 3K). By contrast, it decreased the control and the 0.1 Gy readouts for Endothelin-1 in TICAE and TIME cells (Figures 3G,O,P), and the 0.1 Gy readouts for, IL-6, VCAM-1, PECAM-1, TNF-α, E-Selectin and IL-8 in TIME cells (Figures 3I,M,Q,S,U,X), suggesting baseline/low dose effect of TAT-Gap19. We further evaluated the effect of TAT peptide exposure (100 μm) at later time points and conditions where TAT-Gap 19 demonstrated inhibitory effects in both TICAE and TIME cells (Supplementary Figure 3). TAT peptide did not influence IL-1β, IL-8, MCP-1, VCAM-1, and Endothelin-1 levels at 72 h and 7 days post exposure in TICAE cells. In TIME cells, TAT peptide only increased MCP-1 and VCAM-1 at 72 h, and IL-8 at 7 days post exposure (Supplementary Figure 3).

### TAT-Gap19 Protects From Radiation-Induced Premature Endothelial Senescence

In order to assess premature senescence in TICAE and TIME cells after 0.1 and 5 Gy irradiation and to evaluate the effect of TAT-Gap19, senescence-associated β-galactosidase (SA β-gal) activity was measured at 7, 9, and 14 days after exposure.

**FIGURE 4:**
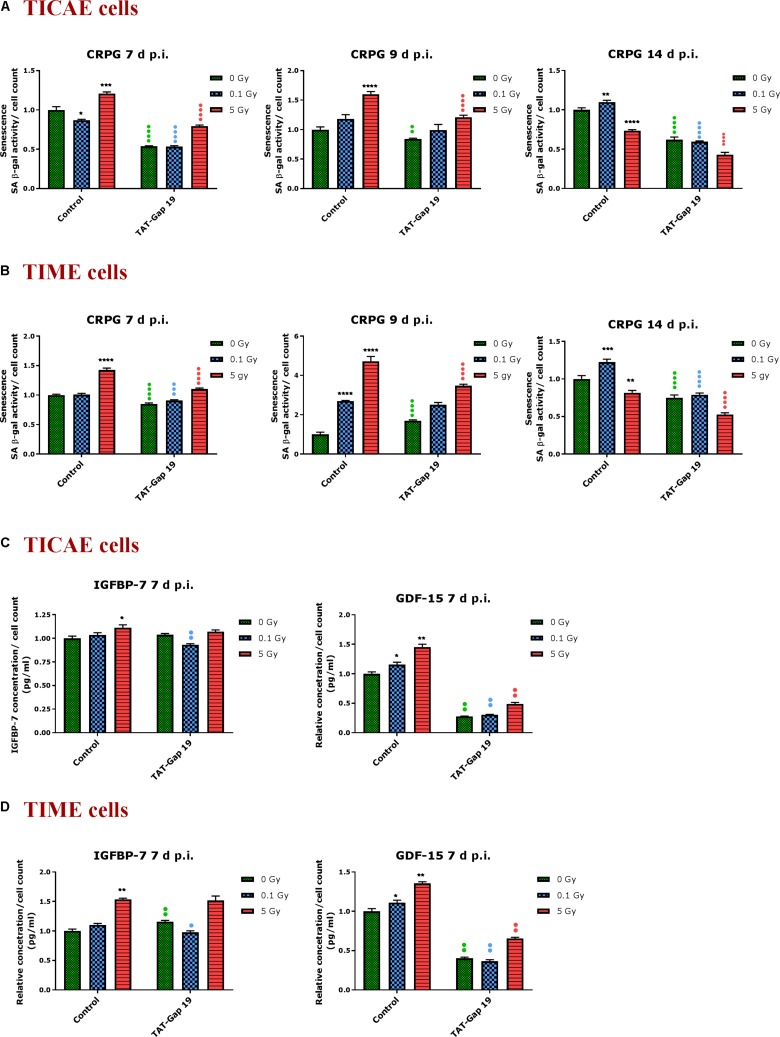
Radiation-induced premature endothelial senescence and effect of TAT-Gap19. Senescence-associated β-galactosidase activity was measured in a CPRG assay at 7, 9, and 14 days after radiation exposure (0.1 and 5 Gy) in **(A)** TICAE cells and **(B)** TIME cells. IGFBP-7 and GDF-15 were assessed in **(C)** TICAE cells and **(D)** TIME cells at 7 days p.i. The values represent the average ± SEM of 16–24 biological replicates in the CPRG assay and of six biological replicates in multiplex-based assays. Statistical significance was analyzed with a non-parametric Mann–Whitney *T*-test. *Indicates statistically significant differences compared to the respective 0 Gy controls. •Indicates statistically significant differences between the TAT-Gap19 group compared to the respective control condition (not treated with TAT-Gap19). */•*p* < 0.05; **/••*p* < 0.01., ***/•••*P* < 0.001, ****/••••*P* < 0.0001.

In TICAE cells, a radiation-induced increase in SA β-gal activity was observed for 5 Gy at 7 and 9 days p.i. By contrast, at the 0.1 Gy 7 days p.i. time point, SA β-gal activity was significantly decreased (Figure 4A). TAT-Gap19 significantly reduced SA β-gal activity at control, 0.1 and 5 Gy conditions for 7 days and at 0 and 5 Gy irradiated conditions for 9 days p.i. (Figure 4A). In TIME cells, a radiation-induced increase in SA β-gal activity was observed for 5 Gy at 7 days p.i. and for 0.1 Gy at 9 days p.i. (Figure 4B). TAT-Gap19 significantly reduced SA β-gal activity in all conditions (0 Gy, 0.1 and 5 Gy) at the 7 days p.i. time point and at the 9 days p.i. time point for 5 Gy; although it unexpectedly increased SA β-gal activity in the control condition in TIME cells at 9 days p.i. (Figure 4B). At 14 days p.i., SA β-gal activity was decreased after 5 Gy exposure but significantly increased after 0.1 Gy exposure in both TICAE and TIME cells, and TAT-Gap19 significantly inhibited these responses (Figures 4A,B).

In order to further assess premature senescence, levels of the Insulin-like Growth Factor-Binding Protein-7 (IGFBP-7) and Growth Differentiation Factor 15 (GDF-15), known to be involved in senescence, were assessed at 7 days after IR exposure using multiplex-based assays. A radiation-induced increase in IGFBP-7 was observed at 5 Gy for TICAE and TIME cells, but this response was not inhibited by TAT-Gap19 (Figures 4C,D). By contrast, 0.1 Gy did not significantly increase IGFBP-7 at 7 days p.i. but TAT-Gap19 acted in a significantly inhibitory way in this condition (Figures 4C,D). The control readout of IGFBP-7 in TIME cells was increased by TAT-Gap19 (Figure 4D). GDF-15 appeared more sensitive to irradiation and was significantly increased in TICAE and TIME cells at both 0.1 and 5 Gy doses at 7 days p.i. TAT-Gap19 significantly inhibited these responses in all treated conditions including the 0 Gy condition (Figures 4C,D).

### Radiation-Induced DNA Damage Is Not Affected by TAT-Gap19

**FIGURE 5:**
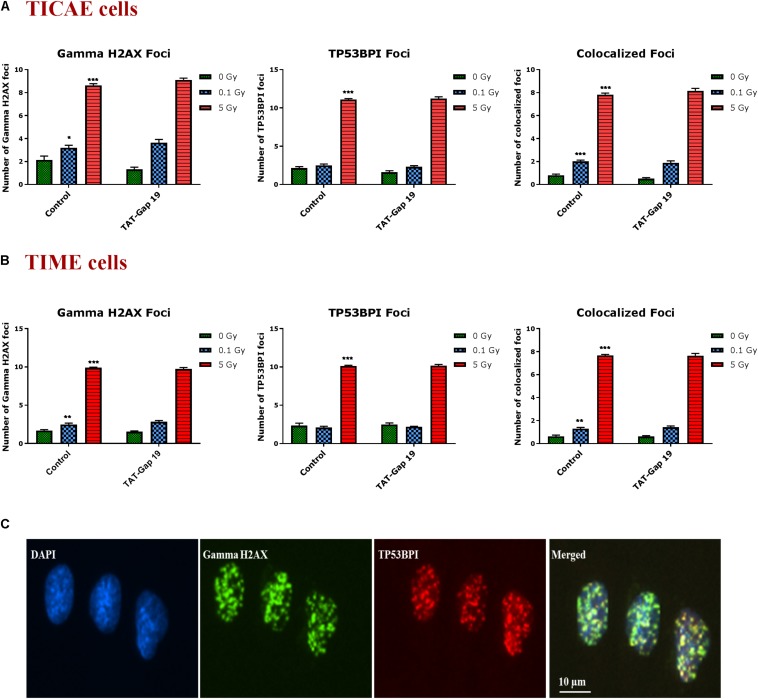
Radiation-induced endothelial DNA damage and effect of TAT-Gap19. Gamma H2AX foci, TP53BPI foci and the colocalized Gamma H2AX/TP53BPI foci were assessed in **(A)** TICAE cells and **(B)** TIME cells at 1 h after 0.1 and 5 Gy of X-ray exposure with or without applying TAT-Gap19. **(C)** Representative images showing gamma H2AX foci (green), TP53BPI foci (red) and colocalized gamma H2AX/TP53BPI foci (yellow) in DAPI stained nuclei of TICAE cells at 5 Gy dose. The values represent the average ± SEM of eight biological replicates; statistical significance was analyzed with a non-parametric Mann–Whitney *T*-test. *Indicates statistically significant differences compared to the respective 0 Gy controls. •Indicates statistically significant differences in the TAT-Gap19 group compared to the corresponding responses in the control group (not treated with TAT-Gap19). */•*p* < 0.05; **/••*p* < 0.01., ***/•••*p* < 0.001.

DNA damage was assessed by immunocytochemical staining for gamma H2AX and TP53BP1 (Figure 5C), DNA double strand break markers (Wang et al., 2014; Kleiner et al., 2015; Baselet et al., 2017), in TICAE and TIME cells after 1 h of X-ray exposure (0.1 and 5 Gy). We furthermore tested the effect of hemichannel inhibition with TAT-Gap19.

In TICAE and TIME cells, radiation induced a significant dose-dependent increase in gamma H2AX and TP53BP1 foci at the 5 Gy dose (Figures 5A,B). The colocalized gamma H2AX/TP53BPI foci also significantly increased in a dose-dependent manner in the irradiated TICAE and TIME cells (Figures 5A,B). These responses were not affected by TAT-Gap19.

## Discussion

**FIGURE 6:**
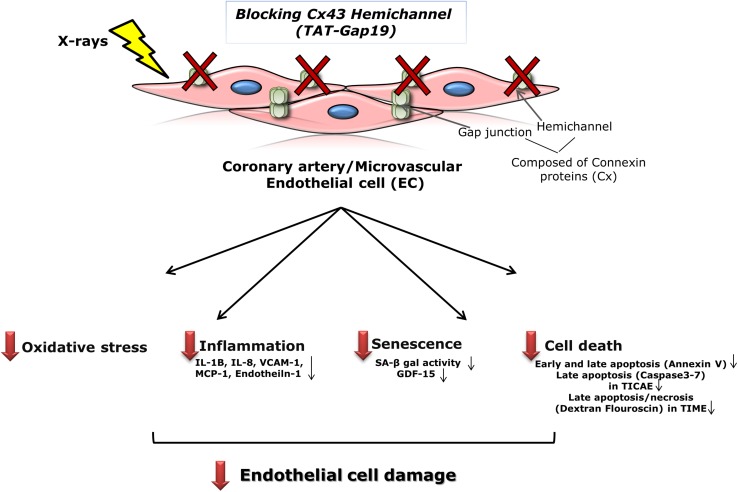
Schematic overview of the experimental findings. Blocking Cx43 hemichannel with TAT-Gap19 significantly reduced oxidative stress, inflammation, senescence and cell death in the irradiated coronary artery and microvascular (TICAE and TIME) endothelial cells, and these responses were mainly observed at the high dose (5 Gy).

Patients treated with thoracic radiotherapy have an increased risk of cardiovascular disease, but the underlying pathophysiology is complex and not fully understood (Yamada et al., 2005; Stewart et al., 2006; Hoving et al., 2008; Shimizu et al., 2010; Icrp et al., 2012; Darby et al., 2013; Kreuzer et al., 2015; Baselet et al., 2016). Here, we aimed to investigate the role of Cx43-based hemichannels in radiation-induced endothelial cell damage, an early marker of atherosclerosis, making use of the peptide inhibitor TAT-Gap19 (Wang et al., 2013b; Delvaeye et al., 2018). We found that this peptide significantly reduced oxidative stress, cell death, key inflammatory cytokines and premature cell senescence induced by X-ray exposure of endothelial cell lines derived from coronary artery and microvascular endothelial cells (Figure 6). Below we discuss these findings in more detail.

A predominant effect of IR exposure is ROS production and oxidative stress (Robbins and Zhao, 2004; Azzam et al., 2012; Yamamori et al., 2012). Accordingly, in TICAE (coronary artery-derived) and TIME cells (microvascular endothelial cells TIME), we found a dose-dependent increase in oxidative stress after 0.1 and 5 Gy of X-ray exposure as measured by CM-H2DCFDA-based live cell imaging approache. The highest oxidative stress response was observed at 45 min p.i. in both cell types, followed by a decline to the 2 and 3 h time points; the latter were, however, still significantly above non-irradiated controls. The decline at 2–3 h may be linked to antioxidant defense reactions of enzymes such as superoxide dismutases (SOD), glutathione, glutathione peroxidases, catalase and others (Robbins and Zhao, 2004; Azzam et al., 2012; Szumiel, 2015). A similar timeline has been observed in primary microvascular endothelial cells exposed to gamma-rays (Laurent et al., 2006). The observed differences between TICAE and TIME cells in the extent of ROS production is possibly related to different radiosensitivity and distinct endothelial properties at different sites of the vascular tree (Aird, 2012; Park et al., 2012). Interestingly, we found that blocking Cx43 hemichannels with TAT-Gap19 significantly reduced ROS production in both cell types at 45 min p.i. Cx43 hemichannel opening has been demonstrated previously in osteocytes in response to oxidative stress, which involved changes of intracellular Ca^2+^ concentration (Kar et al., 2013; Riquelme and Jiang, 2013). Oxidative stress was also reported to induce Cx43 hemichannels opening in fibroblastoid rat mammary tumor cell line (Marshall Cells) which express Cx43 only (Ramachandran et al., 2007). Further downstream, hemichannel opening has been linked to an extracellular depletion of the major antioxidant glutathione, thereby exaggerating oxidative stress in renal tubular epithelial cells (Chi et al., 2016). Along the same line, Cx43 hemichannel inhibition with TAT-Gap 19 increased SOD activity in mice liver treated with thioacetamide to induce liver fibrosis (Crespo Yanguas et al., 2018). We hypothesize that IR exposure induces ROS production in endothelial cells, which opens Cx43 hemichannels and leads to either loss of hemichannel-permeable antioxidants such as glutathione or activates other signaling pathways that lead to the loss of larger antioxidants such as SOD. TAT-Gap19 attenuated this oxidative stress response, but given the important bidirectional relation between [Ca^2+^]i elevation and ROS generation (Yan et al., 2006; Gorlach et al., 2015), it is also possible that hemichannel inhibition directly reduces ROS production by inhibiting Ca^2+^ entry through hemichannels, though further investigations are needed to clarify the involvement of Cx43 hemichannels in intracellular oxidative stress induced by ionizing radiation exposure.

It is well known that oxidative stress is associated with the activation of various cascades including the DNA damage response, apoptosis and inflammatory pathways, thereby amplifying endothelial dysfunction (Elahi et al., 2009; Frey et al., 2009; Soucy et al., 2011; Schramm et al., 2012; Tapio, 2016; Kattoor et al., 2017; Incalza et al., 2018). We observed radiation-induced DNA damage in the two cell types at 1 h p.i., as evidenced by gamma-H2AX and TP53BP1 foci analysis. These findings are consistent with previous studies where a dose-dependent increase in DNA damage was observed in EA.hy926 endothelial hybrid cells, in umbilical vein endothelial cells, as well as in coronary artery endothelial cells in response to X-ray doses in the range of 0.05 to 2 Gy (Rombouts et al., 2013; Baselet et al., 2017), and in microvascular endothelial cells after 3 and 10 Gy of gamma-rays (Laurent et al., 2006). Our findings showed that TAT-Gap19 did not alter this DNA damage response in the irradiated TICAE and TIME cells.

We found that cell death in the two cell types mainly occurred after 5 Gy irradiation, with TICAE cells responding with Annexin V and Caspase 3/7 elevation while TIME cells responded with Annexin V elevation and membrane leakage as indicated by cellular dextran fluorescein positivity. The absence of Caspase 3/7 elevation in TIME cells confirms the findings in primary human microvascular endothelial cells exposed to X-rays in the 2 to 12 Gy dose range (Muschter et al., 2018). Overall, the cell death observed in our study is in line with previous findings in umbilical vein endothelial cells (Rombouts et al., 2013) and microvascular endothelial cells (Langley et al., 1997; Rousseau et al., 2011). Interestingly, we show that TAT-Gap19 significantly reduced cell death in irradiated TICAE and TIME cells. Cx43 hemichannels are known to mediate cell death in various model systems [reviewed in Decrock et al. (2009b)] as well as in cardiomyocytes exposed to hypoxia-reoxygenation or ischemia-reperfusion (Wang et al., 2013b; Gadicherla et al., 2017), by facilitating the passage of soluble factors and molecules such as ATP, ROS and Ca^2+^ (Leist et al., 1997; Li et al., 2001; Hur et al., 2003; Kalvelyte et al., 2003; Rodriguez-Sinovas et al., 2007; Decrock et al., 2009a, b; Saez et al., 2010; Belousov et al., 2017). We (in a previous study) and others reported that IR induces hemichannel-related ATP release (Kang et al., 2008; Ohshima et al., 2012; Ramadan et al., 2019) which may cause intracellular ATP depletion, thereby activating cell death process through necrosis or apoptosis (Leist et al., 1997; Kalvelyte et al., 2003; Decrock et al., 2009a). Hemichannel opening has furthermore been suggested to facilitate ROS entry into the cells eventually leading to cell death (Ramachandran et al., 2007). Moreover, Cx43 hemichannel inhibition with TAT-Gap19 was previously reported to reduce cell death in various model systems (Wang et al., 2013b; Gadicherla et al., 2017), and to have anti-apoptotic activity by increasing the expression of Bcl-2 and decreasing that of Bax (Chen et al., 2019). Our experiment further showed that TAT-Gap19 decreased cell death in the control non-irradiated condition, as observed by an increase in Annexin V and Caspase 3/7 detection in TICAE cells and by an increase in Annexin V and Dextran fluorescein detection at 72 h in TIME cells. This could be explained by Cx43 hemichannels opening in response to several stress stimuli (Ramachandran et al., 2007; De Vuyst et al., 2009; Orellana et al., 2009; Johansen et al., 2011; Meunier et al., 2017), which could be associated with manipulation of cells during experiments or confluent cells in culture, resulting in a basel level of ATP leakage and cell death, counteracted by TAT-Gap19 protection.

Inflammation plays a key role in atherosclerosis development and progression (Libby, 2012). In this study, we showed that radiation induced an increase in atherogenic inflammatory markers (IL-6, MCP-1, PECAM-1, IL-1β, TNF-α, CRP, VCAM-1, E-Selectin, Endothelin-1, IL-8, and PAI-1) mainly at 5 Gy, in both TICAE and TIME cells. Observations in various endothelial cells confirm an elevation of these inflammatory markers mainly at higher irradiation doses (>2 Gy) (Hallahan et al., 1995; Van Der Meeren et al., 1999; Lanza et al., 2007; Scharpfenecker et al., 2009; Kiyohara et al., 2011; Abderrahmani et al., 2012; Haubner et al., 2013; Sievert et al., 2015; Himburg et al., 2016; Baselet et al., 2017). We found that TAT-Gap19 significantly reduced the elevated IL-1β, IL-8, VCAM-1, MCP-1, and Endothelin-1 levels in the two cell lines, at late time points (72 h and 7 days), while it also had early (24 h) inhibitory effects in TIME cells mainly at 5 Gy. In addition, TAT-Gap19 displayed early (24 h) inhibition of TNF-α, E-Selectin, PECAM-1, CRP, and ICAM-1 mainly at 5 Gy but only in TIME cells, indicating that Cx43 hemichannel involvement in the inflammatory response is time and cell line dependent. All cytokines listed are known to be strongly involved in the pathogenesis of radiation-induced atherosclerosis. The cytokine response is triggered by IR/oxidative stress-induced NF-κb signaling and results in endothelial cell activation with subsequent expression of adhesion molecules (Kim et al., 2001; Roedel et al., 2002; Elahi et al., 2009; Liao, 2013; Sievert et al., 2015). IL-6 has been shown to be responsible for propagating downstream inflammatory responses, and expression of adhesion molecules, such as VCAM-1, ICAM-1, PECAM-1, and E-Selectin, on the vascular wall which is known to initiate the recruitment of macrophages and leukocyte from the vasculature, that acts together with MCP-1 and IL-8 to induce infiltration of macrophages into the subendothelial cell layer, leading to the formation of atherosclerotic lesions (Harrington, 2000; Libby et al., 2002; Schuett et al., 2009; Kiyohara et al., 2011; Libby, 2012). Moreover, increased the production of the potent vasoconstrictor peptide Endothelin-1 was linked to endothelial cell dysfunction, as it may decrease endothelial nitric oxide synthase (eNOS) expression, thereby reducing nitric oxide (NO) vasodilatory signaling and it was also reported to activate macrophages (Kinlay et al., 2001; Bousette and Giaid, 2003; Loomis et al., 2005; Napoli et al., 2006; Bohm and Pernow, 2007). Importantly, it is thought that Cx43 hemichannel opening may continue and propagate the inflammatory scene (Retamal et al., 2007; Guo et al., 2016; Li et al., 2018; Saez et al., 2018). In addition, upregulated Cx43 expression often correlates with increased inflammatory responses, while reduced Cx43 expression inhibits inflammation (Mori et al., 2006; Retamal et al., 2007; Cronin et al., 2008; Danesh-Meyer et al., 2008; Tsuchida et al., 2013; Mugisho et al., 2018). TAT-Gap19 has been demonstrated to significantly decrease IL-1β and TNF-α in non-alcoholic fatty liver mouse model (Willebrords et al., 2017). Peptide5, another mimetic peptide-based hemichannel blocker, was reported to reduce TNF-α and IL-1β in rat model of spinal cord injury (O’Carroll et al., 2013). This peptide also reduced inflammation in a rat model of light-induced retinal damage, as assessed by glial fibrillary acidic protein (GFAP) and leukocyte common antigen (CD45) immunohistochemistry (Guo et al., 2016; Mat Nor et al., 2018). A possible mechanism by which Cx43 hemichannels contribute to inflammation is through their regulatory effect on high-mobility group box 1 (HMGB1), which serves as a damage-associated molecular pattern molecule (DAMP) (Li et al., 2018). HMGB1, released by apoptotic cells, induces IL-6, IL-8, and MCP-1 secretion and increases the expression of ICAM-1 and VCAM-1 in endothelial cells (Sun et al., 2013). Furthermore, by releasing ATP, Cx43 hemichannels enhance inflammation through downstream activation of the NLRP3 inflammasome (Mugisho et al., 2018; Mugisho et al., 2019). Cx43 hemichannels may thus act to amplify and perpetuate an initial inflammatory condition. Interestingly, IR induces a rapid and persisting increase in Cx43 gene and protein expression in TICAE and TIME cells (Ramadan et al., 2019), thereby creating pro-inflammatory substrate.

In contrast to the inflammation mitigating effect of TAT-Gap19 on irradiated endothelial cells, the peptide transiently increased some other inflammatory markers at various time points (Supplementary Table 1). TAT-Gap19 is composed of the nonapeptide Gap19, linked to a HIV-derived TAT sequence to promote its membrane permeability (Abudara et al., 2014). It is possible that this HIV-derived membrane translocation sequence may induce pro-inflammatory side effects in the cells. In line with this, alpha-carboxy terminus 1-peptide (αCT1), another Cx43 based peptide containing the antennapedia translocation motif, triggered a transient increase in TNF−α upon topical corneal application (Moore et al., 2014). Here, we report that the TAT translocation motif by itself induces a transient increase in IL-6, MCP-1, IL-1β and CRP in TICAE cells at 24 h p.i. (the time point at which TAT-Gap19 significantly increased all assessed cytokines in TICAE cells). In addition, TAT peptide increased MCP-1 and VCAM-1 only in TIME cells at 72 h post exposure (of note; TAT-Gap19 significantly increased the 0 Gy control and some of the irradiated conditions in these cytokines at 72 h p.i. in TIME cells). Taken this into account, our results indicate that TAT-Gap19 exerts a late inflammation mitigating effect, by reducing the atherogenic inflammatory markers IL-1β, IL-8, VCAM-1, MCP-1, and Endothelin-1, in both TICAE and TIME cells after IR exposure.

Interestingly, inflammation has been reported to contribute to cellular senescence (Freund et al., 2010; Tian and Li, 2014). Premature cell senescence is known to contribute to endothelial dysfunction by stimulation a complex pro-inflammatory response, increasing ROS levelsand it is known to trigger the initiation of cell death by reducing cell repair ability (Collado et al., 2007; Xue et al., 2007; Donato et al., 2015). Our experiments showed an increase in SA-β gal activity in TICAE and TIME cells mainly at 7 and 9 days p.i. This increase in SA-β gal activity was persistent until 14 days p.i. only at 0.1 Gy. For 5 Gy, an unexpected decrease in SA-β gal activity was observed, which may be caused by the frequent medium change for this time point, resulting in washout of senescent and/or apoptotic cells, which in our experiment mainly occurred at the high dose, and replacement by proliferating cells. Premature senescence in TICAE and TIME cells was also apparent from the increase in IGFBP-7 (at 5 Gy) and GDF-15 (at 0.1 and 5 Gy) 7 days p.i. Our results are in line with previous studies, where cellular senescence was observed after low and high doses of IR exposure in different types of endothelial cells, including human coronary artery (Oh et al., 2001; Ungvari et al., 2013; Yentrapalli et al., 2013a, b; Kim et al., 2014; Lowe and Raj, 2014; Baselet et al., 2017). Interestingly, TAT-Gap19 significantly suppressed the radiation-induced increases in SA-β gal activity, mainly at 5 Gy at 7 and 9 days and from 0.1 Gy at 14 days, and GDF-15 levels from 0.1 Gy in TICAE and TIME cells suggesting a role for Cx43 hemichannels. Chronic Cx43 hemichannel opening, as reported in our previous study at 72 h after irradiation in TICAE and TIME cells (Ramadan et al., 2019), may result in ATP leakage that in turn can activate downstream cellular processes including propagation of intercellular Ca^2+^ waves, ROS production, activation of the NLRP3 inflammasome pathway and inflammation (De Bock et al., 2012; Mugisho et al., 2018; Tonkin et al., 2018), which are all known to stimulate premature cellular senescence (Kurz et al., 2004; Acosta et al., 2013; Tchkonia et al., 2013). In addition, senescence can be communicated to neighboring cells in a paracrine manner involving secretion of the senescence-associated secretory phenotype (SASP) and inflammasome activation (Acosta et al., 2013). Presumably, Cx43 hemichannels may perhaps facilitate paracrine senescence communication between cells via its effects on pro-inflammatory parameters like IL-8, MCP-1, and IL-6 which are inhibited by TAT-Gap19 in our study. We further showed that TAT-Gap19 decreased senescence in some of the control non-irradiated conditions. Several stress stimuli might be associated with confluent cells in culture for longer times (7 to 14 days), which may result in a basal level of senescence that can be protected by TAT-Gap19, as shown in our experiment.

In summary, this study demonstrates that TAT-Gap19 mitigates radiation-induced endothelial cell damage by reducing oxidative stress, cell death, pro-inflammatory and pathological factors like IL-1β, IL-8, VCAM-1, MCP-1, and Endothelin-1 and premature senescence. This effect is observed mainly for the 5 Gy dose. Further research is needed to investigate the underlying pathways and molecular mechanisms leading to radiation-induced Cx43 hemichannel opening, including changes in membrane potential and intracellular Ca^2+^, redox status, the surface amount of Cx43 hemichannels and the channel opening probability. Investigating the amount of functional connexin hemichannels at the cell surface may also help in understanding the variable response of TAT-Gap19 in different cell types. In addition, it will be interesting to gather information regarding the downstream effects of Cx43 hemichannel opening that trigger major changes in endothelial function and survival such as unveiling the role of purinergic signaling, intracellular Ca^2+^, ionic balance and intracellular redox status. Other open question that needs to be resolved is what is the effect of other Cx43 hemichannel targeting approaches, such as Gap19 without HIV-derived TAT, siRNA/shRNA or other mimetic peptides, on endothelial cell response after irradiation. This may open perspectives for the establishment of novel therapeutic strategies to protect from secondary radiotherapy side effects in thoracic cancer patients.

## Conclusion

In conclusion, to the best of our knowledge, this is the first study to show the possible involvement of endothelial Cx43 hemichannels in contributing to various radiation-induced processes, such as oxidative stress, cell death, inflammation and premature cell senescence that lead to endothelial activation and dysfunction, which is known as an early marker for atherosclerosis. Therefore, targeting Cx43 hemichannels holds potential to therapeutically protect against radiation-induced endothelial cell damage.

## Data Availability Statement

The raw data supporting the conclusions of this article will be made available by the authors, without undue reservation, to any qualified researcher.

## Author Contributions

RR conducted all the experiments and wrote the manuscript text. EV did a MSc project contributing to inflammatory marker measurement at 48 h and Dextran fluorescein experiments. DA did a MSc project contributing to ROS experiment using Flow Cytometry, inflammatory markers measurements at 24 and 72 h, and apoptosis live cell imaging experiments. IG performed a MSc project contributing to live cell imaging of ROS and DNA damage experiment. DH participated in Calcium measurement experiment which directed us in the discussion. ED, SB, LL, and AA contributed in designing the experiments and supervision of the work. All authors, except the MSc students EV, DA, and IG contributed equally in reviewing the manuscript. All authors read and approved the final version of the manuscript.

## Conflict of Interest

The authors declare that the research was conducted in the absence of any commercial or financial relationships that could be construed as a potential conflict of interest.
