# Supplementary material for: Connexin43 Hemichannel Targeting With TAT-Gap19 Alleviates Radiation-Induced Endothelial Cell Damage
Source: Front Pharmacol. 2020 Mar 5;11:212. doi: 10.3389/fphar.2020.00212 (PMC7066501; doi:10.3389/fphar.2020.00212)
Supplement: Supplementary file 1 [file Image_1.pdf]

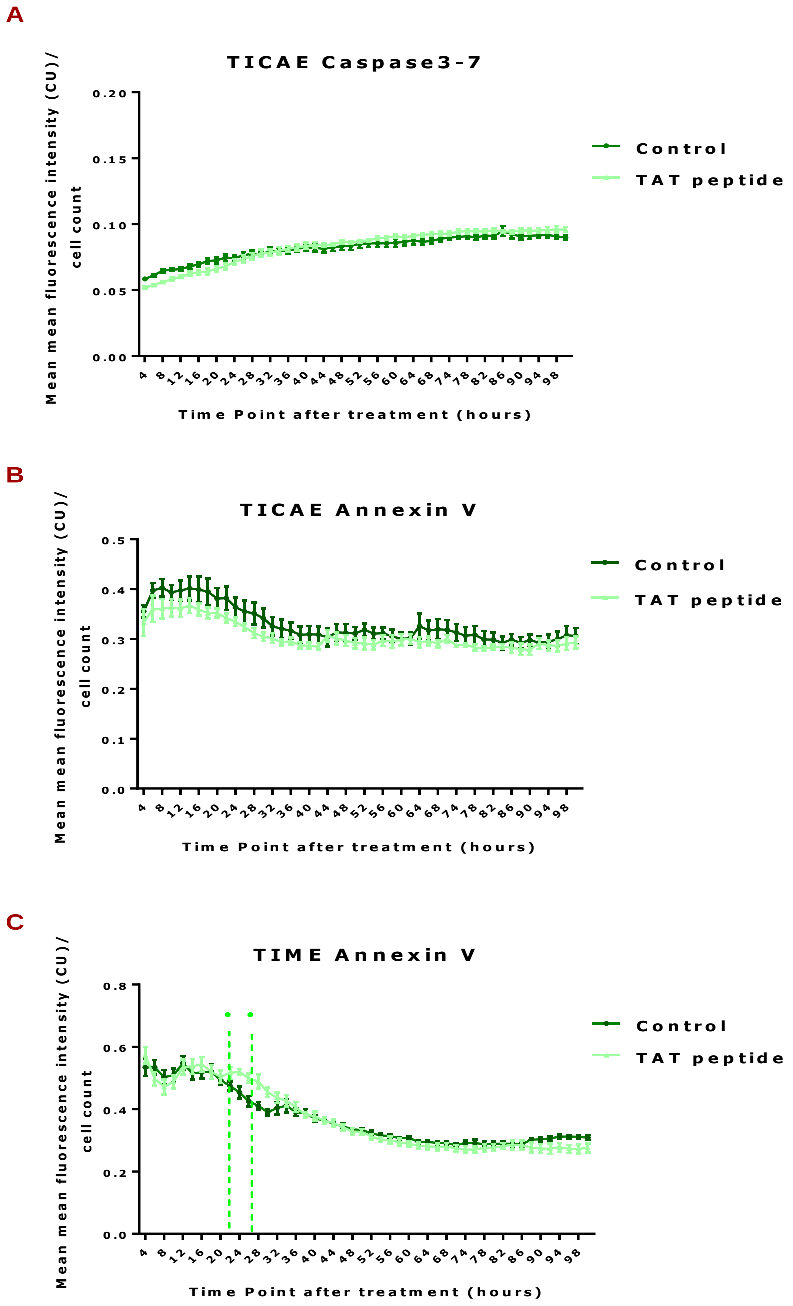

Supplementary Figure 1: The effect of TAT peptide on cell death in TICAE and TIME cells. Caspase 3/7 activity was assessed in TICAE cell and Annexin V was assessed in TICAE and TIME cells from 4 h until 100 h of 100 µl of TAT peptide exposure using Incucyte live cell imaging of the mean fluorescence intensity measured in Calibrated Unit (CU) that takes in account acquisition time and fluorescent intensity. (A and B) No significant changes in Caspase 3/7 activity and Annexin V signal were observed for TAT peptide compared to untreated control in TICAE cells. (C) In TIME cells, there was a slight increase in Annexin V signals with TAT peptide only from 24h until 30h after exposure compared to the untreated control. Data were analyzed with a nonparametric two-way ANOVA followed by Tukey test. The values represent the average ± SEM of 8 biological replicates.
