# Supplementary material for: Connexin43 Hemichannel Targeting With TAT-Gap19 Alleviates Radiation-Induced Endothelial Cell Damage
Source: Front Pharmacol. 2020 Mar 5;11:212. doi: 10.3389/fphar.2020.00212 (PMC7066501; doi:10.3389/fphar.2020.00212)
Supplement: Supplementary file 2 [file Image_2.pdf]

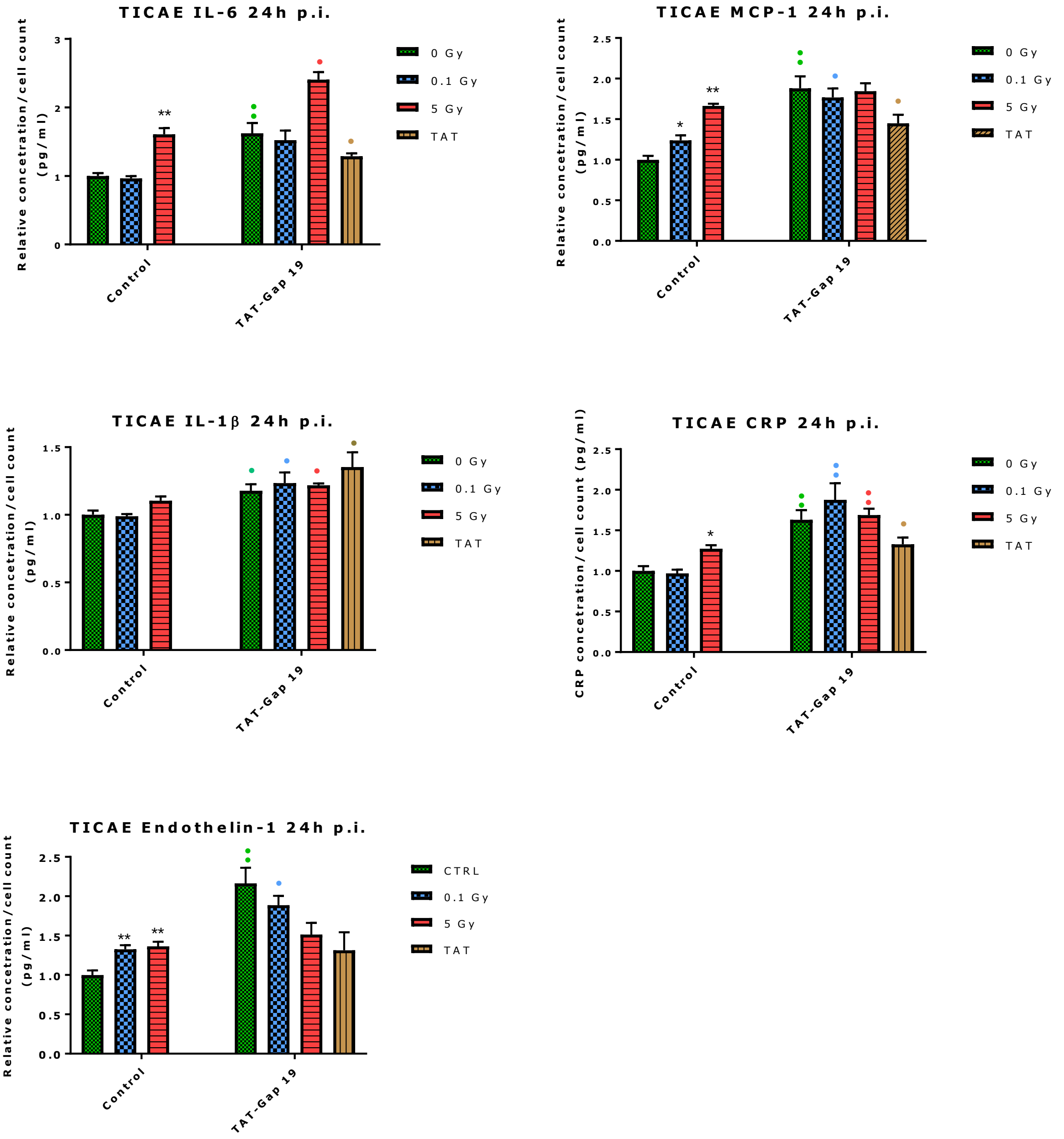

Supplementary Figure 2: The effect of radiation exposure, TAT-Gap19 and TAT alone on IL-6, MCP-1, IL-1β, CRP and Endothelin -1 in TICAE cells at 24h post exposure. Data were analyzed with a nonparametric Mann-Whitney T-test. The values represent the average ± SEM of 5 biological replicates. * indicates the statistical differences compared to the respective 0 Gy controls. • indicate the statistical difference compared to the respective radiation dose of the control conditions (un-treated with TAT-Gap19). */•: $p<0.05$; **/••: $p<0.01$. IL-6, interleukin 6; IL-1β, Interleukin 1 beta; MCP-1, Monocyte chemoattractant protein 1; CRP, C-reactive protein.
