# Supplementary material for: Connexin43 Hemichannel Targeting With TAT-Gap19 Alleviates Radiation-Induced Endothelial Cell Damage
Source: Front Pharmacol. 2020 Mar 5;11:212. doi: 10.3389/fphar.2020.00212 (PMC7066501; doi:10.3389/fphar.2020.00212)
Supplement: Supplementary file 3 [file Image_3.pdf]

TICAE cells

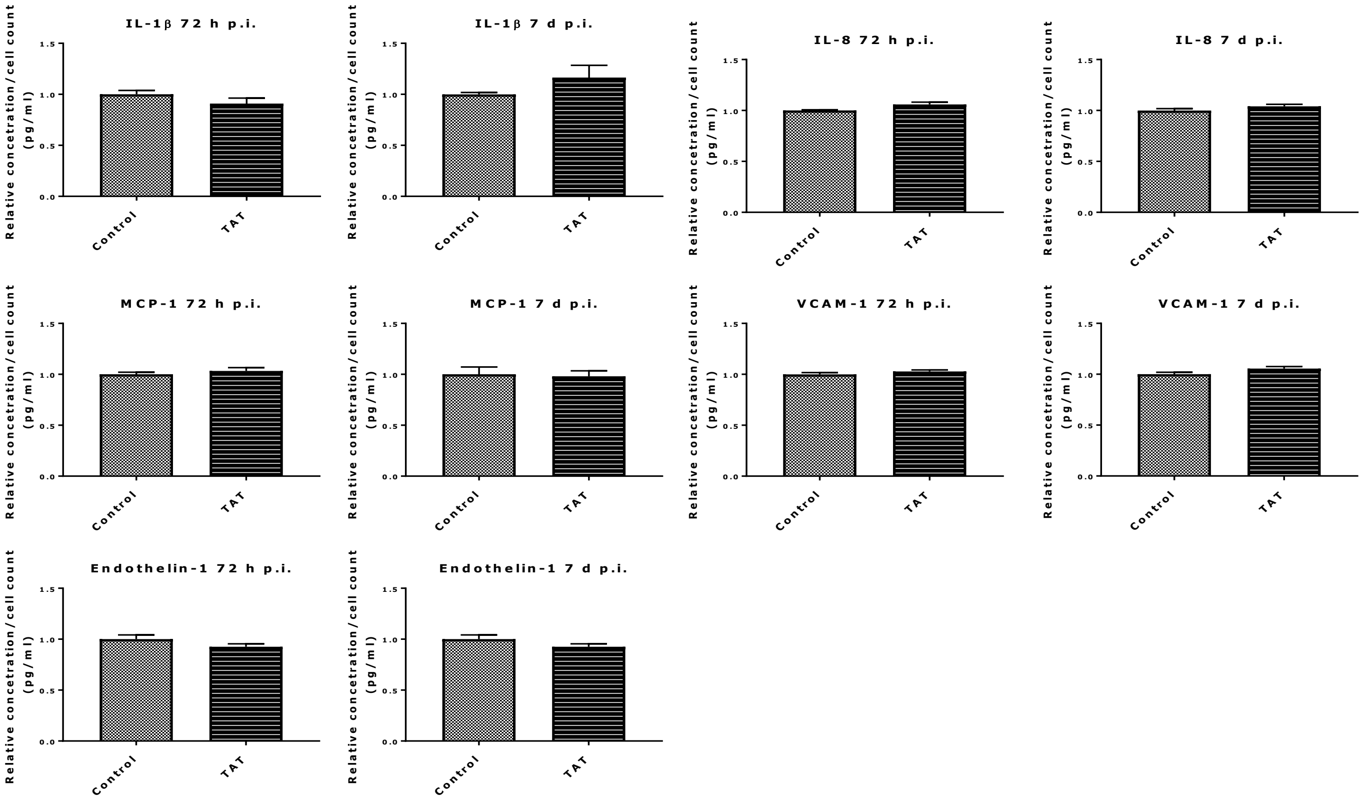

TIME cells

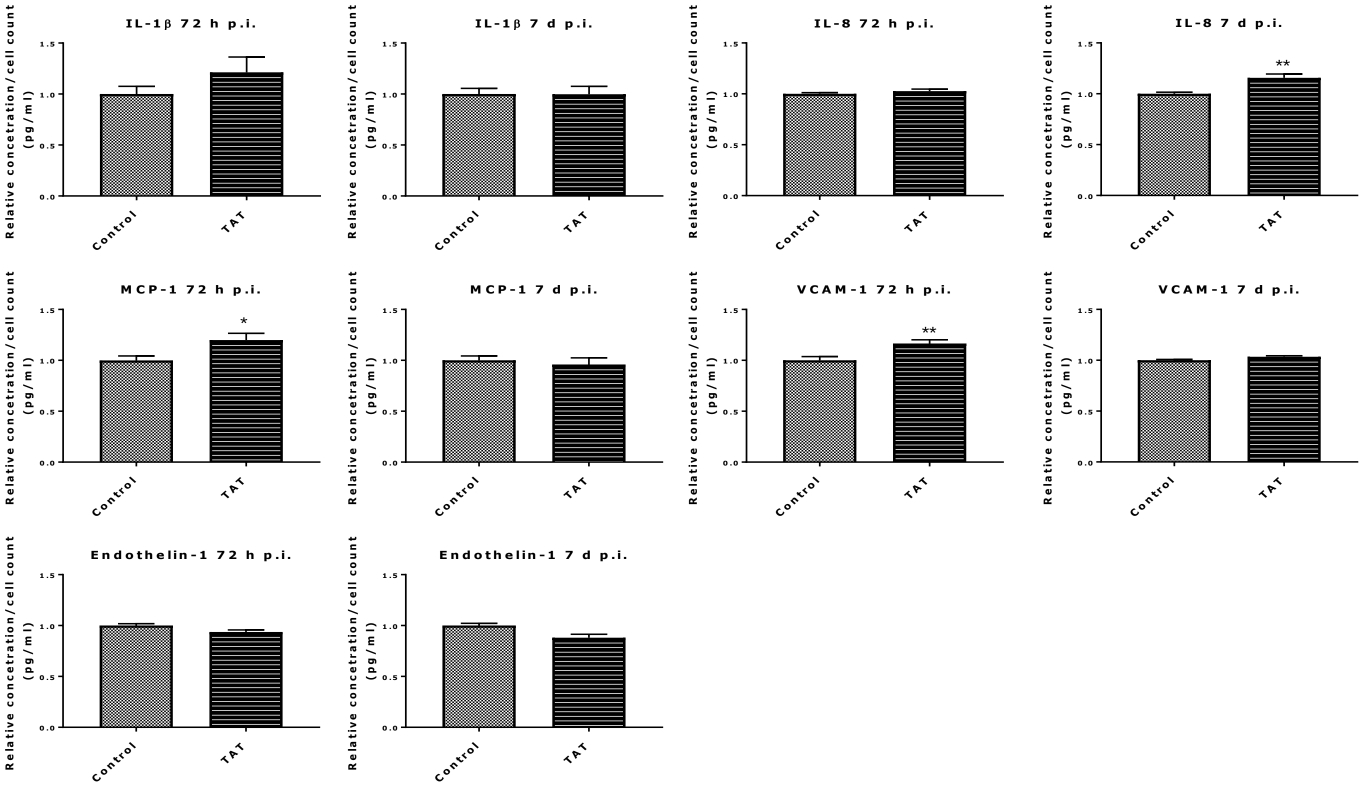

Supplementary Figure 3: The effect of TAT peptide exposure on IL-1β , IL-8, MCP-1, VCAM-1 and Endothelin-1 in TICAE and TIME cells at 72 h and 7 d post exposure. Data were analyzed with a nonparametric Mann-Whitney T-test. The values represent the average ± SEM of 6 biological replicates. * indicates the statistical differences compared to the control. *: $p<0.05$; **: $p<0.01$. IL-8, interleukin 8; IL-1β, Interleukin 1 beta; MCP-1, Monocyte chemoattractant protein 1; VCAM-1, Vascular cell adhesion protein 1.
