# Supplementary material for: Connexin43 Hemichannel Targeting With TAT-Gap19 Alleviates Radiation-Induced Endothelial Cell Damage
Source: Front Pharmacol. 2020 Mar 5;11:212. doi: 10.3389/fphar.2020.00212 (PMC7066501; doi:10.3389/fphar.2020.00212)
Supplement: Supplementary file 4 [file Table_1.pdf]

Supplementary Table 1: The effect of radiation exposure and TAT-Gap19 on different atherosclerosis inflammatory markers at 24 h, 48 h, 72 h and 7 d post exposure in TICAE and TIME cells. The effect of 0.1 Gy and 5 Gy of X-rays exposure was compared to 0 Gy controls, while 0 Gy, 0.1 Gy and 5 Gy TAT-Gap19 was compared to the respective radiation dose of the control (IR only) conditions.

Cytokines	TIME point after IR	TICAE cells						TIME cells					
		IR			IR+ TAT-Gap19			IR			IR+TAT-Gap19		
		0 Gy	0.1 Gy	5 Gy	0 Gy	0.1 Gy	5 Gy	0 Gy	0.1 Gy	5 Gy	0 Gy	0.1 Gy	5 Gy
IL-6	24 h												
MCP-1													
PCAM-1													
IL-1β													
TNF-α													
CRP													
VCAM-1													
E-Selectin													
ICAM-1													
Endothelin-1													
IL-6	48 h												
MCP-1													
PCAM-1													
IL-1β													
TNF-α													
CRP													
VCAM-1													
IL-6	72 h												
MCP-1													
PECAM-1													
IL-1β													
TNF-α													
CRP													
VCAM-1													
E-Selectin													
IL-8													
ICAM-1													
Endothelin-1													
PAI-1													
IL-6	7 d												
MCP-1													
IL-1β													
VCAM-1													
IL-8													
Endothelin-1													
IGFBP-7													
GDF-15													

No significant changes
A significant increase, when $p<0.05$
A significant increase, when $p< 0.01$
A significant decrease, when $p<0.05$
A significant decrease, when $p<0.01$
